# Stress factors among physiotherapy students at a university in Gauteng, South Africa

**DOI:** 10.4102/hsag.v30i0.2803

**Published:** 2025-01-31

**Authors:** Zander I. Collins, Chané Siepker, Kelebogile J. Ralehlaka, Boitshoko C. Molefe, Tiisetso C. Phala, Simphiwe A. Vilankulu, Makwena M. Sibuyi, Thembelihle S. Ntuli

**Affiliations:** 1 Department of Physiotherapy, School of Health Sciences, Sefako Makgatho Health Sciences University, Tshwane, South Africa; 2 Department of Statistical Science, School of Science and Technology, Sefako Makgatho Health Sciences University, Tshwane, South Africa

**Keywords:** physiotherapy students, perceived stress levels, stressors, psychological morbidities, emotional self efficacy

## Abstract

**Background:**

Physiotherapy students are highly susceptible to experiencing burnout during training. Prolonged exposure to stressful environments predisposes students to psychological morbidities.

**Aim:**

The study assessed the perceived level of stress and stressors among a cohort of final-year physiotherapy students, registered at a medical university in Gauteng province, South Africa.

**Setting:**

The study was conducted at a single medical university in Gauteng province with registered final-year undergraduate physiotherapy students for the 2024 academic year.

**Methods:**

A descriptive cross-sectional study with a total population sample of 42 physiotherapy students. Data were collected through an online anonymised self-administered questionnaire. The Cronbach alpha coefficients for each subscale were 0.99 for physical, 0.72 for interpersonal relationships, 0.85 for academic and 0.81 for environment. Statistical analysis was conducted using IBM Corp’s Statistical Package for the Social Sciences (SPSS) Software version 25.

**Results:**

The median age of participants was 22 years (iqr ± 3.8) with 69.0% of them being females. Perceived stress levels ranged between low (*n* = 24, 57.14%) to moderate (*n* = 18, 42.85%) with females being at risk, as measured by the Student Stress Inventory. Academic and environmental factors contributed to perceived moderate to severe stress levels.

**Conclusion:**

The study found the severity of stress levels to be slightly below what has been reported in other studies. However, attention needs to be directed to female students to develop emotional self-efficacy skills. Early identification of psychosomatic disorders could indicate the need for interventions to prevent psychological and physiological morbidities.

**Contribution:**

The study adds to the body of knowledge pertaining to the mental health of students at higher institutions of learning.

## Introduction

Students in healthcare science programmes such as dentistry, medicine and pharmacy have been reported to suffer high levels of stress (George & Joseph 2018; Shakthivel et al. 2017) during their academic years. Stress is subjective and depends on the cognitive appraisal of the individual. Studies have investigated the severity of stress among university physiotherapy students in Asian countries (Alias, Mustafa & Hamzah 2020; Patel & Hadiya 2020; Syed, Ali & Khan 2018; Wong & Chapman 2023) and West Africa (Ezekiel 2015) reported moderate levels of stress. According to the authors’ knowledge, the literature is limited that investigated the severity of stress among physiotherapy students in South African universities. A study conducted among physiotherapy students at the University of the Free State during their clinical years reported moderate levels of stress (Van Vuuren, Bodenstein & Nel 2018). Literature reports that uncontrolled chronic stress predisposes students to more serious mental health disorders (Atkinson 2020). For this reason, it is essential to investigate the severity of stress and be able to identify stressors to encourage appropriate help-seeking behaviours among physiotherapy students.

The physiotherapy programme at the undergraduate level runs for a duration of 4 years (Health Professions Council of South Africa 2023) on a full-time basis apart from a few institutions in Pakistan and Nigeria offering the programme over a 5-year period (Higher Education Commission in Pakistan 2024; Nigerian Society of Physiotherapy 2024). The undergraduate physiotherapy programme is highly demanding because of its composition of theoretical, practical and clinical components. The first and second years of the physiotherapy programme are referred to as preclinical years implying that students learn more theory about a plethora of health conditions. The third-year and fourth-year programmes are referred to as the clinical years implying students spend more time at clinical sites interacting with patients from communities in primary and secondary levels of care (HPCSA 2023). Clinical training is more likely to heighten physiotherapy student stress levels as it has been reported to contribute to the perceived high level of stress among nursing and medical students (Van Vuuren et al. 2018).

In the life of students, various authors (Patel & Hadiya 2020; Stallman & Hurst 2016; Van Vuuren et al. 2018; Webber et al. 2022), using different tools (General Health Questionnaire 21, Depression Anxiety Stress Scale 21, Occupational Therapy Student Stress Scale and the University Stress Scale and the Student Stress Inventory) showed commonality pertaining to stressors in students. These stressors are mostly organised into categories being academic, physiological, interpersonal relationships and environmental stressors. Most commonly, students are overwhelmed with fear of failure because of the work overload with submission deadlines. The inability to control this fear results in excessive worry, headaches and fatigue (Alias et al. 2020; Licayan et al. 2021). On the interpersonal level, maintaining good relationships with their peers is beneficial although they find it difficult to engage lectures and family when they need support (Patel & Hadiya 2020). Students are also burdened with the desire to fulfil parents’ expectations (Zheng, Zhang & Ran 2023), which makes them want to improve their results (Nagle & Sharma 2018; Subramani & Venkatachalam 2019). In a study in Eritrea environmental stressors involved inadequate campus facilities, crowding, air pollution and lack of time management (Yikealo, Yemane & Karvinen 2018).

Physiotherapy students are reported to be at risk of experiencing burnout while training (Brooke et al. 2020). Burnout is a consequence of being exposed to stressful environments for long periods. The feeling of being burned out is a growing problem, especially at the workplace restricting productivity but increasing psychological morbidities in individuals (Calitz 2022; Demerouti et al. 2021). Lack of early identification of stressors predisposes physiotherapy students to long-term health conditions that will lead to increased student attrition and low graduation rates (Fitzgerald, McCabe & Molyneux 2020; Hegarty, Le & Wilkins 20213). Therefore, investigating the perceived level of stress would provide an indication of the severity of stress students experience amid the demanding academic life (Alias et al. 2020). The main purpose of this study was to assess the level of perceived stress and contributing factors among a cohort of final-year physiotherapy students at a medical university in Gauteng province, South Africa.

## Research methods and design

### Study design

A cross-sectional, quantitative and descriptive study was conducted with students from the medical university in Gauteng province, South Africa (Siedlecki 2020). The data collection phase was conducted over a period of 1 month, in May 2024.

### Study population and setting

The population included final-year registered students in the Department of Physiotherapy. The study population was chosen in response to the increasing number of students seeking assistance from campus psychosocial support services. During the 2024 academic year, 47 students registered for the final year programme.

### Sampling technique and sample size

Considering the population size of approximately 47 registered final-year undergraduate physiotherapy students, non-probability sampling, utilising a total population sampling technique, was chosen because of its suitability for small populations of fewer than 100 individuals with specific characteristics of interest (Harefa et al. 2023). Therefore, excluding the five student researchers, the study included all (*N* = 42) the final year registered physiotherapy students.

### Data collection

An online self-administered questionnaire was created using Microsoft Forms, accompanied by a consent form outlining the objectives of the study. A link to the survey was shared with participants via a class WhatsApp group. Students signed in with the institutional email addresses. The questionnaire included questions on demographic characteristics being sex, age, race, nationality, accommodation, final year repeater, number of years in the university, having child(ren), being pregnant and marital status. Following this section, there were 40 items from the Student Stress Inventory (Arip et al. 2020), divided into four subscales: physical stress, stress from interpersonal relationships, academic stress and environmental stress.

Each item had four response options: 1 ‘*never*’, 2 ‘*somewhat frequent*’, 3 ‘*frequent*’ and 4 ‘*always*’. Stress levels were measured for each participant by summing the scores of all items to obtain an overall score. An overall score between 40 and 80 indicated a low stress level, 81 and 121 indicated a moderate stress level and 122 and 160 indicated a severe stress level.

To ascertain the factors that contribute to the level of stress, the items were summed up within each subscale for each participant. Therefore, subscale scores falling within the range of 10 – 18 indicate a low degree of stress, scores between 19 and 29 suggest a moderate level of stress and scores between 30 and 40 indicate a severe level of stress. The Cronbach alpha coefficients for each subscale were as follows: 0.99 for physical, 0.72 for interpersonal relationship, 0.85 for academic and 0.81 for environment.

### Data analysis

The statistical analysis was conducted using IBM Corp’s Statistical Package for the Social Sciences (SPSS) Software version 25. The data were examined using both descriptive and analytical methods and presented as the mean, median and percentages. The Fisher exact test was used to assess the association between categorical variables, while the Mann–Whitney U test was applied to examine the relationship between a continuous variable and a level of experienced stress. The Spearman rank correlation was employed to assess the relationship between two continuous variables. A *p*-value less than 0.05 was considered to have statistical significance.

### Ethical considerations

The Sefako Makgatho Health Sciences Research Ethics Committee granted ethical authorisation (Reference: SMUREC/H/101/2024:UG). Permission to conduct the study was obtained from both the Dean and the Head of the Department of Physiotherapy. Participation in the study was entirely voluntary and individuals provided their written informed consent electronically before completing the survey. All respondents received information about crisis and student counselling services, as well as details on where to seek emergency care if they felt uncomfortable while filling out the survey. All information was securely saved and anonymised as participants responded to the online survey.

## Results

### Demographic characteristics of participants

A total of 42 physiotherapy final-year students participated in this study. Their median age was 22 years with an interquartile range of 3.8 years. Approximately 73.8% of the participants were less than 25 years of age. Most of the participants were (69.0%) females and 90.5% were Africans. Nearly all (97.6%) of the participants were unmarried and 83.3% were living in the university’s residence. None of the participants were repeating their final year, and none were pregnant, apart from one who had a child. Table 1 provides a detailed description of the participants.

**TABLE 1:** Relationship between level of perceived stress and demographics.

Variables	*n*	Level of perceived stress				*p*
		Low		Moderate		
		*n*	%	*n*	%	
**Age (years)**						
< 25	31	14	45.2	17	54.8	0.012
≥ 25	11	10	90.9	1	9.1	-
**Sex**						
Male	13	9	69.2	4	30.8	0.333
Female	29	15	51.7	14	48.3	-

### Level of perceived stress

In the study involving 42 students, 24 (57.1%) displayed low levels of perceived stress and 18 (42.9%) exhibited moderate levels. Notably, no students reported experiencing severe stress symptoms. The association between the level of perceived stress and demographics is shown in Table 1. Participants who were 25 years and younger had a considerably higher likelihood of experiencing moderate symptoms of perceived stress compared to those who were 25 years and older (54.8% versus 9.1%, *p* < 0.05). No significant relationship between levels of perceived stress and sex (*p* > 0.05) was found, but females (48.3%) were experiencing more moderate symptoms of perceived stress than males (30.8%).

Table 2 indicates that, among the various factors influencing stress levels, academic and environmental factors were identified as contributing to moderate to severe stress in the current study. Table 3 revealed no statistically significant relationship between sex and the cause of stress. However, females exhibited moderate levels of physical and interpersonal stress, as well as moderate to severe levels of academic and environmental stress, in comparison to males.

**TABLE 2:** Overall self-reported stress level per sub-scale (*N* = 42).

Variables	*n*	Low stress		Moderate stress		Severe stress	
		*n*	%	*n*	%	*n*	%
Physical	40	24	57.1	18	42.9	0	0.0
Interpersonal relationship	40	24	57.1	18	42.9	0	0.0
Academic	40	12	28.6	27	64.3	3	7.1
Environment	40	13	31.0	24	57.1	5	11.9

**TABLE 3:** Association between sex with source of stress.

Subscales	Sex				*p*
	Male		Female		
	*n*	%	*n*	%	
**Physical**					
Low stress	10	76.9	14	48.3	0.104
Moderate stress	3	23.1	15	51.7	-
**Interpersonal relationship**					
Low stress	8	61.5	16	55.2	0.748
Moderate stress	5	38.5	13	44.8	-
**Academic**					
Low stress	6	46.2	6	20.7	0.190
Moderate stress	7	53.8	20	68.9	-
Severe stress	0	0.0	3	10.3	-
**Environmental**					
Low stress	5	38.5	8	27.6	0.127
Moderate stress	5	38.5	19	65.5	-
Severe stress	3	23.0	2	6.9	-

The analysis of the connections between age and different factors related to stress revealed a weak negative relationship. However, this correlation was not statistically significant (*p* > 0.05). Table 4 illustrates the specific findings about the correlation between age and the sources of stress.

**TABLE 4:** Correlation between the age of the participants and the source of stress.

Items	Variables	1	2	3	4	5
1	Age	1	-	-	-	-
2	Physical	−0.065	1	-	-	-
3	Interpersonal relationship	−0.230	0.188	1	-	-
4	Academic	−0.289	0.317*	0.641*	1	-
5	Environmental	−0.009	0.190	0.348*	0.088	1

* , *p* < 0.05.

Table 5 displays the specific items within each subscale. The primary physical stressors include extreme exhaustion, excessive concern and back pain. When these sources were further analysed by sex, it became apparent that women were significantly more likely than men to experience excessive worry (*p* = 0.0325). The stressors in interpersonal relationships included parental expectations, challenges in expressing feelings to parents and the inability to meet parental aspirations. However, there was no notable disparity in the sources of stress between males and females in this regard (*p* > 0.05).

**TABLE 5:** Total score for individual items in each subscale.

Items	Mean	s.d.	Median	IQR
**Physical**				
Headaches	3.8	12.1	2	1
Back pain	4.4	14.0	2	1
Sleep problem	3.9	12.4	2	1
Difficulty breathing	2.9	8.2	2	1
Excessive worry	4.5	14.5	2	1
Stomach pain and/or nausea	3.4	11.0	2	1
Extreme fatigue	4.9	15.7	2	1
Sweaty hands	2.4	7.8	1	0
Frequent cold, flu and/or fever	3.0	9.7	1	1
Drastic weight loss	2.5	7.9	1	0
**Interpersonal relationship**				
I feel guilty if I fail to fulfil my parent’s hope	2.4	1.1	2	20
It is difficult for me to express emotions to my parents	2.3	1.1	2	2
My parents wish only for my success	3.6	0.7	4	0
It is difficult to get academic help from friends	1.5	0.9	1	1
Often have a problem with close friends, boyfriend or girlfriend	1.6	0.9	1	1
My friends did not care about me	1.4	0.7	1	0
I feel ashamed to meet the lecturer	1.7	0.9	1.5	1
My families are not supportive	1.3	0.8	1	0
My lecturers are not supportive	1.7	0.9	2	1
I have difficulty communicating with the university’s management	1.2	0.6	1	1
**Academic**				
I am having trouble mastering a difficult lesson	1.8	0.7	2	1
I find it difficult to juggle time between study and social activity	2.2	0.9	2	1
I feel nervous about delivering the class presentation	2.8	1.20	3	2
I feel stressed as the submission deadline neared	2.7	1.0	3	2
I am not ready to sit for the examination	2.4	0.9	2	1
I always come late to lectures or classes	1.6	0.6	2	1
I lost interest towards academic	1.9	0.9	2	1
I did not understand what the lecturers had been teaching	1.9	0.8	2	0
I give up on difficult subjects and tasks	1.6	0.7	1.5	1
My academic results were unsatisfactory	2.3	0.7	2	1
**Environmental**				
I have a transportation problem at my university	1.4	0.8	1	1
Life at a hostel or residential house is uncomfortable	1.6	0.9	1	1
Surrounding noise distracted me	2.1	1.0	2	2
Air pollution makes me uncomfortable	2.2	1.2	2	2
I am not confident about the safety of the hostel or residential	1.6	0.7	1.5	1
Disorganised life bothers me	2.8	0.9	3	2
I feel frustrated with the inadequate campus facilities	2.6	1.0	2.5	2
Crowding makes me feel uneasy going outside	2.6	1.1	2	2
Waited in a long line made me feel uneasy	2.5	1.1	2	2
I feel scared of being at an insecure place	2.7	1.1	3	2

IQR, interquartile range, s.d., standard deviation.

Academic stressors encompass nervousness about presenting in class and stress related to submission deadlines and examination periods, with females significantly more likely to experience stress during class presentations compared to their male counterparts (*p* = 0.0004). The environmental factors that contribute to stress include a lack of organisation in one’s lifestyle, insufficient facilities on campus and residing in regions that are both unsafe and overcrowded. Furthermore, there was no notable disparity between males and females with regard to these stresses (*p* > 0.05).

## Discussion

This study examines the stress levels of final-year physiotherapy students at a South African university, which predominantly educates African students in the medical field. While the research is confined to one university, the results indicate an increase in the representation of black students in physiotherapy within higher education and training institutions, contrasting with their historical underrepresentation (Bunting & Cloete 2016; Cobbing 2021). The majority of the study’s participants were female, a finding consistent with a retrospective review of the HPCSA database records from 1938 to 2018, which indicated that most registered physiotherapists were female (Louw et al. 2021). Conversely, other studies in African countries have shown a male predominance (Temesgen et al. 2021), while some have reported an equal distribution between genders (Mbada et al. 2021).

The median age of participants in this study was marginally greater than that reported for physiotherapy students in Nigeria, as indicated by Mbada et al. (2021). Concurrently, a study by Mahlangu, Mokoena and Ntuli (2024), which examined stress levels among undergraduate and postgraduate students at the School of Sciences and Technology within the same South African university, also reported a median age that was slightly above our results. In consistent with the results of the previous study that assessed the prevalence of perceived stress among university students, most of the participants in this study were unmarried (Alsaleem et al. 2021). Universities have effectively leveraged student accommodation as a strategic asset for fostering academic success, aligning with the social integration model (Xulu 2019). The study reveals that a significant majority, 83.33%, of participants resided in university housing.

### Overall stress level

The overall level of self-reported stress experienced by final-year physiotherapy students in the current study ranged between low (*n* = 24, 57.14%) and moderate (*n* = 18, 42.85%) scores. These findings are almost similar to the study investigating stress levels among undergraduate physiotherapy students in India using a similar data collection tool (Patel & Hadiya 2020). The similarity in the overall level of stress can be attributed to commonalities in the demographic characteristics and the duration of the undergraduate physiotherapy programme being 4 years. However, a higher self-reported stress was related to modules being semesterised (Alias et al. 2020) as opposed to being year modules. This could have contributed to the low levels of self-reported stress reported from this current cohort of university students as their modules are not semesterised, having had a longer time to complete modules. Similarly, in Pakistan and Nigeria, although the undergraduate physiotherapy programme is 5 years, students experienced a moderate level of stress, slightly above average at 53% (Syed et al. 2018). In addition, most of these students in the current study did not have family responsibilities and were housed in university student accommodation. Over and above, the resilience of students to manage stress, the university has positioned itself strategically to be student centred. Mahlangu et al. (2024) believed students having access to psychosocial support services in the university and their level of maturity contributed to students in their study reporting overall low levels of stress. Both these factors could have contributed to the reported low levels of stress in the current study. Although there were no statistically significant differences in the level of perceived stress between female and male students, females were more at risk according to the Student Stress Inventory (Arip et al. 2020). This finding was consistent with the study conducted among healthcare sciences undergraduate students in Ethiopia (Awoke et al. 2021). Increased cortisol hormone in females was attributable to higher perceived stress than in males (Faresjö et al. 2014).

### Stress factors

Undergraduate physiotherapy students reported moderate stress levels across all four sub-scales of the Student Stress Inventory. However, most of the stressors were reported within the academic and environmental sub-scales. These findings concurred with the findings of the study by Patel and Hadiya (2020) and Yikealo et al (2018). Stressors ranked high by students within this study were similarly found to concur with other studies conducted among undergraduate physiotherapy students from various universities in Malaysia (Alias et al. 2020) and Nigeria (Ezekiel 2015), Singapore (Wong & Chapman 2023). In most undergraduate dental students, inactive lifestyles were attributable to the high frequency of students experiencing headaches, excessive worry and constant fatigue (Tangade et al. 2011). Students from the above-mentioned study adopted inactive lifestyles as they spent more of their time on academic workload (Shamsuddin et al. 2013).

The academic workload stems from students having to conduct presentations and prepare for examinations. Students also often have the pressure of fulfilling their parents’ expectations and wanting to improve their results as suggested in the study by Nagle and Sharma (2018) and Subramani and Venkatachalam (2019). In a study investigating whether academic stress and test anxiety were affected by parental expectations and regulatory emotional self-efficacy, a significant positive correlation was found to exist between academic stress and test anxiety. However, parental expectations were negatively correlated with academic stress (Zheng et al. 2023).

The current cohort of students reported stress from environmental factors. The level of stress among undergraduate students in India showed a significant positive correlation with environmental factors (Patel & Hay 2020). This finding is also consistent with findings from research among non-health sciences students such as those from Eritrea Institute of Technology, where it was found that crowding, unsafe and inadequate facilities were attributable to environmental stressors among undergraduate students (Yikealo et al. 2018).

### Limitations

The study did not include the first-, second- and third-year physiotherapy undergraduate students because of limited time in conducting the study. Thus, the results are not representative of all the physiotherapy undergraduate students. The study could have also tested for anxiety and depression symptoms as chronic stress predisposes students to mental health disorders. In follow-up studies, focus group discussions are suggested to explore in-depth contributing factors to student stress levels and mitigating factors.

### Recommendations

It is crucial to assess the severity of stress early so that students can receive professional help in managing stress. A review of the curriculum, teaching pedagogies and assessment methods including spacing of examinations could reduce the academic pressure. Where applicable, financial resources need to be allocated to improve the living conditions of students in residential areas.

## Conclusion

The 2024 cohort of final-year physiotherapy students at a medical university in Gauteng province reported low to moderate levels of stress. The study flags female students to be at risk of developing psychological morbidities because of their inherent approach to stress. Furthermore, the study was able to identify the main source of stress to be emanating from the academic and environmental subscales of the Student Stress Inventory. It is important for lecturers and students to become aware of any early experience of psychosomatic disorders and to refer them for counselling services. It may also facilitate early prevention strategies to screen students at all year levels of the physiotherapy programme once per semester for headaches, fatigue, sleeplessness and chronic pain. This information could assist the department in developing individual support plans and monitoring changes post-management by relevant professionals. While presentations, submitting assignments and writing examinations are part of assessment methods, students need to be supported to develop emotional self-efficacy. In this way, students will have better control over the academic pressure. Participation in physical activities needs to be integrated into the calendar. It may be a good idea for the department to propose designating days for sports, which may facilitate interactions with peers and students with lecturers. These interactions could serve as positive support structures which can reduce stress levels among physiotherapy students. Students’ residential areas are also learning environments where students get to study and prepare for examinations. The conditions of the infrastructure, availability of Internet, water and electricity (to mention a few) need to be improved to create a conducive learning environment for better academic outcomes. This study also raises the need for strengthening of the inter-departmental collaboration at the university. The Department of Physiotherapy should work closely with departments concerned with student residence, counselling, sports and recreation to reduce student stress levels.
